# MultiPassMerger: Automated data processing for multipass cyclic ion mobility HDX‐MS

**DOI:** 10.1002/pro.70168

**Published:** 2025-05-24

**Authors:** Vanessa Duerr, Damon Griffiths, Argyris Politis

**Affiliations:** ^1^ Faculty of Biology, Medicine and Health, School of Biological Sciences The University of Manchester Manchester UK; ^2^ Manchester Institute of Biotechnology The University of Manchester Manchester UK; ^3^ Institute for Bioinnovation Biomedical Sciences Research Center “Alexander Fleming” Vari Greece

**Keywords:** cIM‐MS, cyclic ion mobility, HDMS^E^, HDX, hydrogen/deuterium exchange, integral membrane proteins, multipass, peptide mapping

## Abstract

Hydrogen/deuterium exchange‐mass spectrometry (HDX‐MS) is a powerful tool for studying protein structure and dynamics. As a bottom‐up LC‐MS technique, its success largely depends on peptide identifications made by peptide mapping prior to HDX measurements. We previously demonstrated that combining peptide mapping results from complementary single‐ and multipass cyclic ion mobility‐mass spectrometry (cIM‐MS) experiments, an approach we term “multi‐sequence” cIM‐MS, can enhance HDX‐MS by increasing peptide identifications. However, this approach required labor‐intensive, manual handling of the acquired data, including lengthy optimization of drift time (DT) versus DT full width half maximum (FWHM) trendlines during peak detection processing to combat cyclic wrap‐around effects. Here, we present MultiPassMerger, an open‐source software tool that automates the processing, merging, and filtering of single‐ and multipass cIM‐MS peptide mapping data. MultiPassMerger was validated through re‐analysis of several model proteins. Its automated capabilities enabled better optimization of DT versus DT FWHM trendlines on a protein‐specific basis, enhancing peptide identification relative to manually optimized trendlines. Beyond automating our previous approach, MultiPassMerger also introduces a novel “multi‐trendline” processing method, involving iterative processing using multiple trendlines and merging of results to better sample ions across the DT versus DT FWHM distribution. Using MultiPassMerger with both multi‐sequence and multi‐trendline strategies increased peptide identifications up to 392% relative to SYNAPT G2‐Si with linear ion mobility and 102% relative to use of single‐pass cIM‐MS alone. Consequently, MultiPassMerger can enhance peptide mapping and makes this approach practical and more accessible to the wider HDX‐MS community. MultiPassMerger is available as a downloadable Windows executable at https://politislab.uk/multipassmerger.

## 1 INTRODUCTION

HDX‐MS is being increasingly used to study protein higher‐order structure (Masson et al. 2019). The principle of HDX‐MS is to measure mass increases associated with the isotopic exchange of amide hydrogens to deuterons along the polypeptide backbone (Zhang and Smith 1993). This process is time dependent, with the rate of exchange in folded proteins being primarily determined by their amide hydrogen bonding status and solvent accessibility (Vinciauskaite and Masson 2023). As such, HDX‐MS can report on several key protein characteristics, including conformational dynamics (Trabjerg et al. 2018) and folding pathways (Englander and Mayne 2014), as well as protein interactions with proteins (Mandell et al. 1998), lipids (Martens et al. 2018), nucleic acids (Ahmad et al. 2021), and small molecules (Chalmers et al. 2011). In a traditional HDX‐MS experiment (Masson et al. 2019), proteins are first incubated in a deuterated buffer to induce HDX, followed by reaction quenching by decreasing the temperature and pH to 0–1°C and 2.5, respectively. Proteins then undergo digestion via an on‐line column containing an immobilized acid‐active protease. The generated peptides are then separated by LC, followed by a MS measurement. By comparing deuterated peptide masses with their non‐deuterated control equivalents, this “bottom‐up” LC‐MS approach allows deuterium uptake to be measured at peptide level resolution and, thus, localized to specific structural regions of the protein(s). Consequently, the quality of bottom‐up HDX‐MS data is largely dependent on the extent of successful peptide identifications (IDs) made during non‐deuterated peptide mapping experiments prior to the HDX measurement. Whereas higher sequence coverage provides broader information across the protein sequence length, higher redundancy (the number of unique peptides covering each amide position) enhances analytical rigor and improves the resolution of deuterium uptake measurements by leveraging data from overlapping peptides (James et al. 2022; Mayne et al. 2011; Mullahoo et al. 2020; Nirudodhi et al. 2017).

For peptide identification, most HDX‐MS platforms employ MS instruments capable of data‐dependent or data‐independent acquisition (DIA) (James et al. 2022). One such example is MS^E^, a DIA‐based approach originally developed by Waters Corporation (Bateman et al. 2002; Law and Lim 2013). During MS^E^ acquisition, the MS instrument collision energy (CE) dynamically switches between low‐ and high‐energy states without quadrupolar mass selection to simultaneously generate alternating spectra containing all precursor and product ions detected throughout the LC‐MS experiment. The resulting data can then be processed using three algorithms—Apex3D, Peptide3D, and IDENTITY^E^—within Waters Corporation's ProteinLynx Global SERVER (PLGS) software package. These perform peak detection and charge/isotope deconvolution, followed by matching of precursor ions with their associated products and database searching against the target protein(s) sequence (Law and Lim 2013). Owing to the high complexity of the DIA spectra, the MS^E^ approach benefits greatly from retention time (RT)‐assisted alignment of precursor and product ions, which can increase the number and confidence of matches made.

One historical limitation of the HDX‐MS method is the need to keep LC separations short and under quench conditions. A failure to do so can result in excessive deuterium/hydrogen back‐exchange and, thus, a loss of information content generated at the labelling step (Walters et al. 2012). This, in turn, limits available LC‐MS peak capacity, which often results in insufficient peptide IDs owing to peptide co‐elution and MS spectral overlap. As a result, challenging targets, such as integral membrane proteins (IMPs) or large multi‐subunit complexes with high unique sequence complexity (Engen and Komives 2020), often require lengthy and laborious optimization processes to achieve the peptide IDs required for sufficient data quality (Möller et al. 2019; Toporowska et al. 2024; Zhang et al. 2010).

Several approaches have been adopted to alleviate this issue (Anderson and Hudgens 2023; Hamuro and Coales 2018; Hansen and Politis 2020; Möller et al. 2019; Peterle et al. 2023; Rincon Pabon et al. 2024; Venable et al. 2012). One example is ion mobility (IM), a gas‐phase technique that separates ions based on their differential mobilities through an inert buffer gas under the influence of an electric field (Dodds and Baker 2019). The time taken for ions to traverse the IM cell, known as their drift time (DT) or arrival time, occurs on millisecond timescales, thereby allowing IM to increase LC‐MS peak capacity without detrimentally increasing the length of the experiment. The widespread use of IM in LC‐MS was initially driven by the commercialization of the SYNAPT quadrupole time‐of‐flight (QTOF) instruments by Waters Corporation. These instruments contain a traveling‐wave IM spectrometry (TWIMS) cell of linear geometry positioned in between trap and transfer cells capable of performing analyte fragmentation (Pringle et al. 2007). Ion DT profiles are recorded by synchronizing TOF acquisition with gated ion release from the trap, where each gate pulse triggers 200 TOF acceleration pushes and DT is measured by the specific push (or bin) in which the ion is detected. By performing post‐TWIMS fragmentation throughout MS^E^ (referred to as HDMS^E^), peptide identification can be further enhanced via DT‐assisted alignment in addition to RT‐alignment (Law and Lim 2013; Shliaha et al. 2013), as precursor ions and their associated products should have identical DT (Ibrahim et al. 2010). Moreover, IM improves spectral deconvolution and monitoring of peptide isotopic distributions by generating processed MS spectra based on their DT extraction. IM has previously been shown to enhance HDX‐MS analyses (Cryar et al. 2017; Griffiths et al. 2024; Iacob et al. 2008).

Despite the increased LC‐MS peak capacity provided by IM, the increasing complexity of samples, such in situ IMPs, is driving a need for instrumentation with greater resolving power (Javed et al. 2023). The SELECT SERIES Cyclic IM spectrometer with cyclic ion mobility (cIM) technology by Waters Corporation provides a unique means to tackle this issue (Eldrid et al. 2019; Giles et al. 2019; Ujma et al. 2019). While architecturally similar to the SYNAPT, this instrument contains a TWIMS cell of cyclical geometry that permits “dialing up” of IM resolution by extended separations across multiple device passes; known as “multipass” separations. We previously demonstrated the potential of this technology to enhance bottom‐up HDX‐MS applications (Griffiths et al. 2024). When analyzing model IMPs, utilization of a “multi‐sequence” approach, involving contiguous cIM separations under both single‐ and multipass sequences during HDMS^E^ peptide mapping, resulted in both common and unique peptide populations. Combining results from single‐ and multipass cIM‐HDMS^E^ increased peptide ID up to 222% relative to linear geometry IM‐MS and up to 37% relative to use of single‐pass cIM alone. In addition, we also introduced an alternative PLGS‐based data processing strategy for multipass cIM‐HDMS^E^ peptide mapping data (Griffiths et al. 2024). This approach used user‐defined and optimized DT versus DT FWHM trendlines to apply more appropriate kernel smoothing to IM peak features prior to peak detection by the Apex3D algorithm. This adjustment was essential, as the multipass cIM‐HDMS^E^ datasets exhibited a loss of positive linear correlation in DT versus DT FWHM owing to cyclic “wrap‐around” (Breen et al. 2022). This phenomenon compromised peptide IDs when using trendlines auto‐calculated by the default linear regression model in Apex3D, which can normally be used to determine appropriate kernel smoothers for each IM peak feature. Cyclic warp‐around has been described previously (Breen et al. 2022; Giles et al. 2019), and a more in‐depth description of its impact on peptide ID and mitigation via optimized trendlines can be found in Data S1, Supporting Information.

**FIGURE 1:**
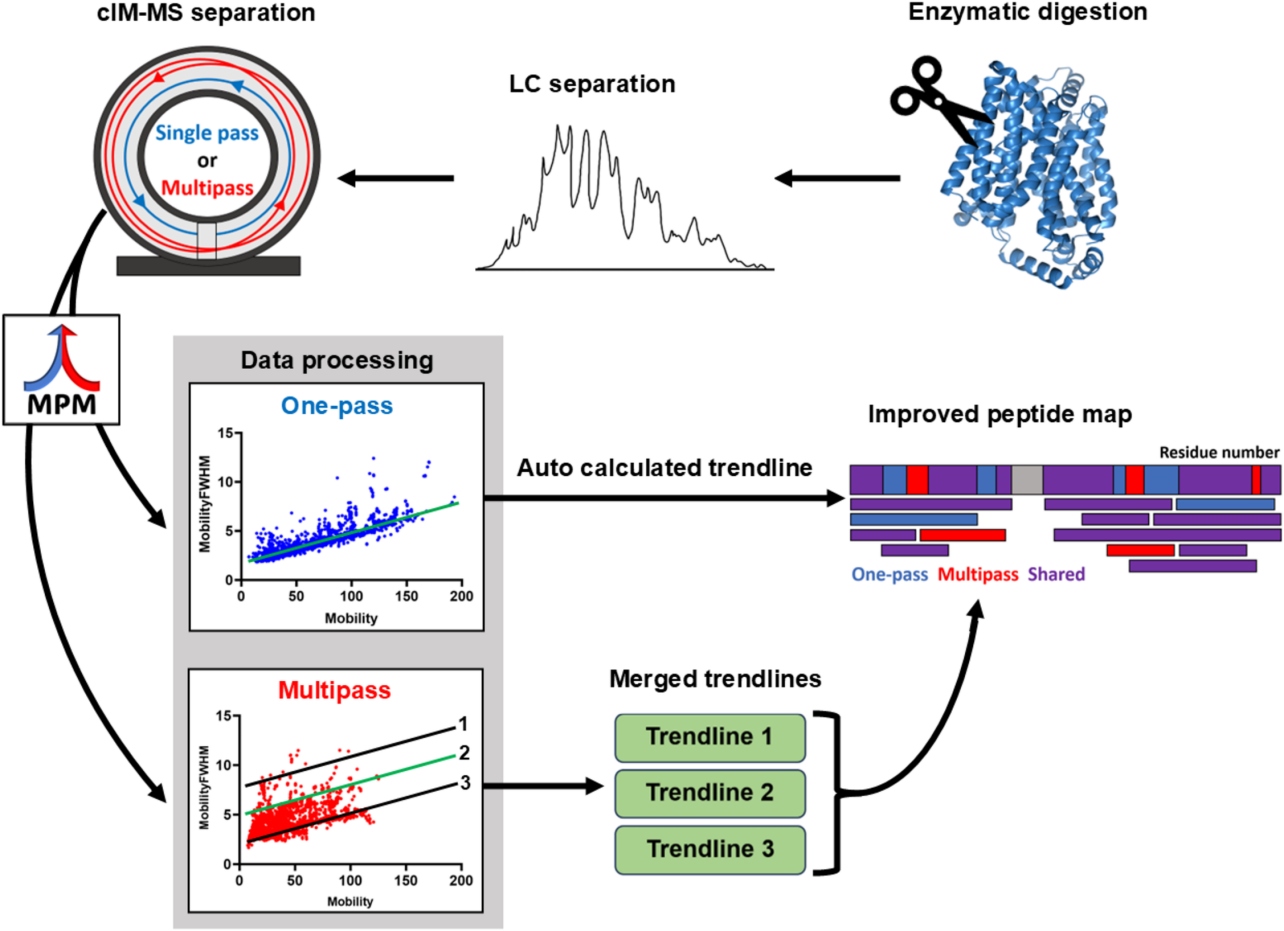
Schematic of the MultiPassMerger workflow. A target protein undergoes enzymatic digestion (e.g., pepsin column) and RP‐LC separation in a classic HDMS^E^ peptide mapping experiment. The cIM‐MS instrument acquires HDMS^E^ data using single‐ and multipass cIM sequences. Single‐pass data is processed with an auto‐calculated trendline, while multipass data uses custom‐set trendlines. Merging these datasets enhances peptide mapping quality, improving subsequent HDX‐MS analyses.

Despite its effectiveness, this data processing approach required hours of laborious manual input. This included iterative processing using different DT versus DT FWHM trendlines, filtering each resulting dataset to identify the best performing trendline(s), and merging the results from single‐pass and multipass cIM‐HDMS^E^ experiments. Here, we present MultiPassMerger, a software tool for the automation of multi‐sequence cIM‐HDMS^E^ peptide mapping data processing (Figure 1). MultiPassMerger provides a user‐friendly graphical user interface that can operate the PLGS ion search algorithms for iterative processing of multipass cIM data using a list of user‐defined trendlines (and auto‐calculated trendlines for single‐pass cIM), filter multipass cIM peptide ID lists to identify the top performing trendline(s), and pool common and unique peptides from single‐ and multipass cIM‐HDMS^E^ experiments to generate larger peptide ID lists for subsequent HDX‐MS experiments. In addition to automating our previously published workflow (Griffiths et al. 2024), MultiPassMerger also permits pooling of peptide IDs made across all of the different DT versus DT FWHM trendline variants applied. We demonstrate the effectiveness of this “multi‐trendline” approach by reanalyzing previously published data from two model detergent‐solubilized IMPs: XylE and smoothened receptor (SMO) (Griffiths et al. 2024). Using multi‐sequence cIM‐HDMS^E^ with multi‐trendline processing, MultiPassMerger achieved peptide ID increases of up to 102% compared to standalone single‐pass cIM with auto‐calculated trendlines, and up to 392% compared to linear geometry TWIMS‐MS. Furthermore, these results significantly outperform previous results obtained using multi‐sequence cIM‐HDMS^E^ using a single optimized trendline (Griffiths et al. 2024). As such, MultiPassMerger represents a notable advancement in HDX‐MS methodology, increasing the information content and resolution of existing cIM‐HDMS^E^ and HDX‐MS data without additional manual effort.

## 2 IMPLEMENTATION

Prior to processing via MultiPassMerger, users must first acquire two LC‐cIM‐HDMS^E^ peptide mapping datasets of the target protein(s) using a SELECT SERIES Cyclic IM spectrometer; one dataset with a single‐pass cIM separation (1–2 ms) and the other with a multipass cIM separation (≥3 ms) (Breen et al. 2022). 18 ms multipass cIM separations were applied here, but optimal separation times will likely be protein‐ and/or instrument parameter‐dependent. Furthermore, it is essential that single‐ and multipass cIM‐HDMS^E^ experiments are performed with identical LC parameters and have comparable RT profiles so that the precursor ions of unique peptides identified during multipass cIM peptide mapping can be successfully monitored in subsequent HDX‐MS experiments acquired using single‐pass cIM‐MS, and vice versa. MultiPassMerger for windows operating systems can be downloaded free of charge via the Politis group website (https://politislab.uk/multipassmerger), which also contains an interactive quick‐start guide, or via GitHub (https://github.com/vanessaduerr/MultiPassMerger), which contains the open access source code. As MultiPassMerger uses the PLGS ion search algorithms, a PLGS (Waters, Wilmslow, UK) license and installation are required. Lastly, the user may choose to manually curate peptide mass spectra, which can be done via DynamX (Waters, Wilmslow, UK) or similar software.

### 2.1 Automation of previous workflow: Multi‐sequence cIM‐MS with optimized trendline data processing

#### 2.1.1 Step 1: Process data

**FIGURE 2:**
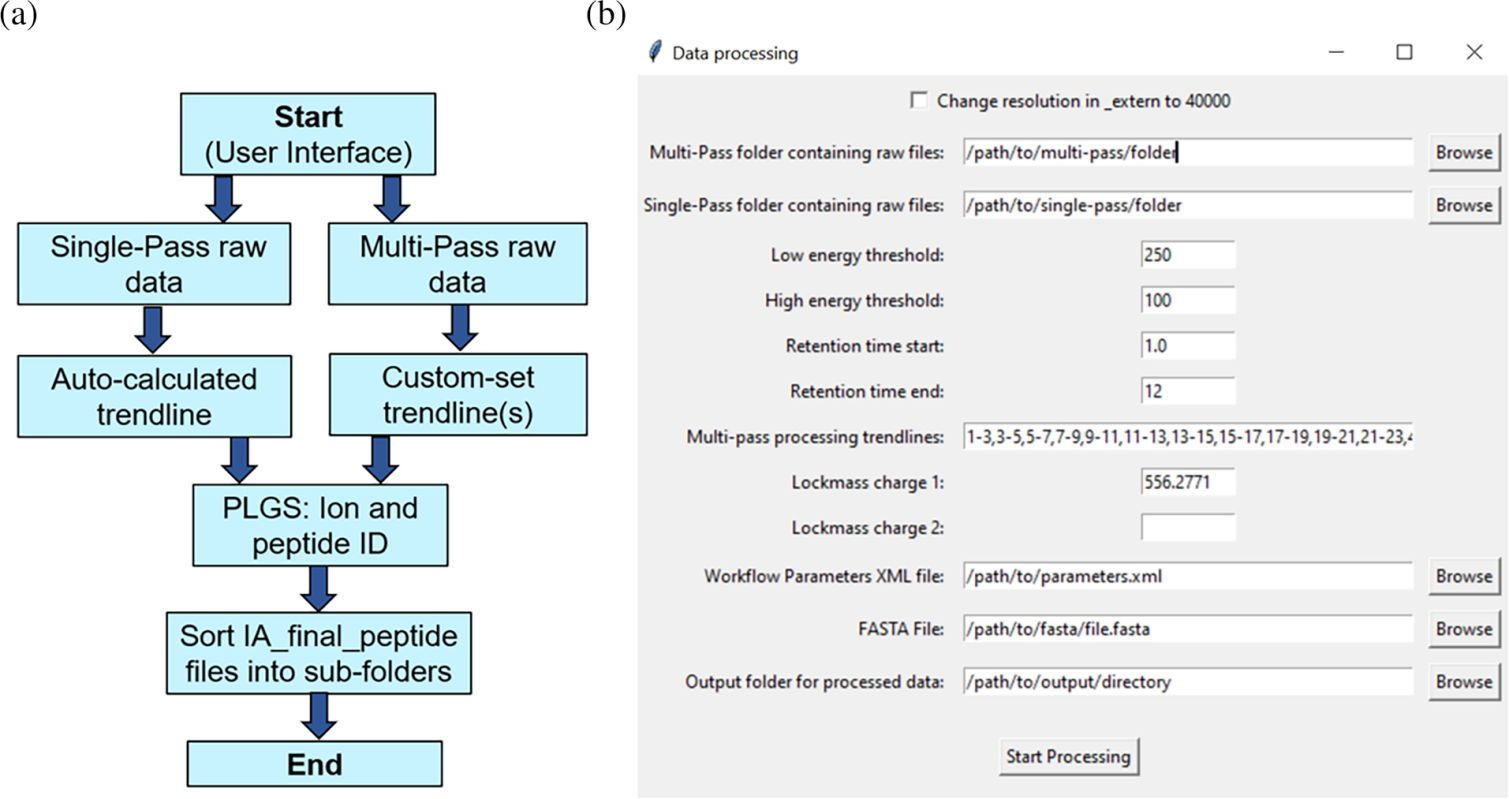
Overview of data processing function in MultiPassMerger. (a) The code logic and (b) interface of the processing step of MultiPassMerger. The user selects different input folders which contain single‐pass and multipass raw data files, which is then processed using an auto‐calculated (for single‐pass) or one or more custom‐set (for multipass) data smoothening trendlines. The PLGS ion and peptide search algorithm is then used to identify the peptides. The finished files containing the peptide IDs are then sorted either into a single‐pass folder (named 1_1) or various multi‐pass folders (named according to the custom‐set trendlines that were used for processing).

After data acquisition, the user opens the “Step 1: Process Data” function via the main MultiPassMerger interface (Figure 2). The user selects two different input folders containing the raw data files from the single‐ and multipass cIM‐HDMS^E^ peptide mapping experiments, respectively (Figure 2b). For the multipass cIM‐HDMS^E^ data, various DT versus DT FWHM trendlines can be manually set by typing in each of the desired trendlines separated by commas in the “Multipass processing trendlines” section. Trendlines are inputted using notation we previously established (Griffiths et al. 2024), where the driftFWHM‐start and ‐end input arguments (which define the drift FWHM at DT bin 0 and 200, respectively) are inputted in hyphen separated format (i.e., 11–13 = driftFWHMstart of 11 and driftFWHMend of 13). Manual trendline setting via driftFWHM‐start and ‐end is explained further in Data S1. For the single‐pass data, an auto‐calculated trendline is generated via Apex3D using its in‐built linear regression model. The user must also provide a FASTA file containing the target protein(s) sequences, and a workflow parameter XML file that can be exported from PLGS (instructions are available on the Politis group website: https://politislab.uk/multipassmerger). Additional processing parameters including low and high energy count thresholds, RT start and end values, and lock mass *m*/*z* can be specified to suit user requirements. Finally, after defining an output folder, the data can be processed. Using the information provided, MultiPassMerger writes and executes a Windows batch file that accesses the PLGS executables (Apex3D, Peptide3D, and IADBs), thereby allowing it to iteratively process the data using the user‐specified trendlines (Figure 2a). The resulting peptide ID lists are then saved and sorted into sub‐folders that are named corresponding to the trendline that was applied (e.g., 11–13 will be 11_13); apart from the single‐pass data which is always “1_1.” The time taken to complete processing is variable and dependent on the available computing power and the size/complexity of the raw files analyzed. For most samples, we recommend using high performance computing, if available, and/or running the data processing function overnight to minimize wait times.

#### 2.1.2 Optional: Best trendline identifier

**FIGURE 3:**
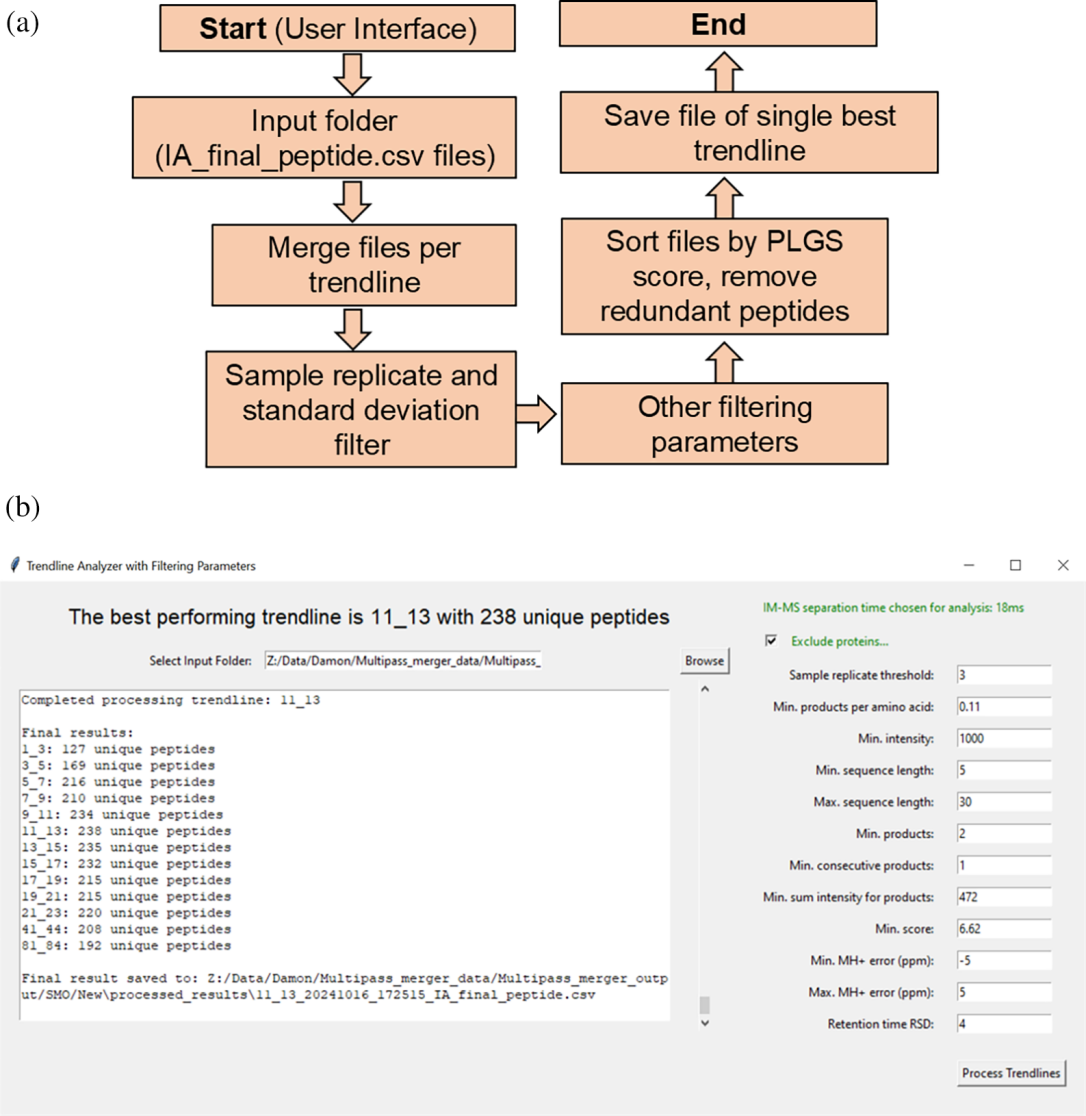
Overview of the best trendline identifier function. The code logic (a) and interface (b) for “Optional: Best Trendline Identifier.” This tool allows the user to identify the best performing trendline(s) for multipass data. Peptide lists are merged per trendline, and the results are filtered according to user‐defined parameters. Peptides are then ranked by PLGS score, and the best scoring unique IDs are kept. The interface displays how many unique peptides were found in each trendline dataset.

After data processing, the resulting peptide ID lists from multipass cIM‐HDMS^E^ peptide mapping can be assessed using the “Optional: Best Trendline Identifier” function (Figure 3). This tool allows users to filter the results generated from each trendline to predict which performed best. These threshold parameters include a sample replicate threshold, minimum products per amino acid, minimum intensity, minimum and maximum peptide sequence length, minimum matched products, minimum consecutive products, minimum sum intensity of products, minimum PLGS score, MH+ ppm error, and RT relative standard deviation (RT‐RSD), (Figure 3b). Here, thresholds based on previously optimized values have been applied (Sørensen and Salbo 2018). Once started, the tool first merges the replicate peptide ID lists within each individual trendline subfolder, followed by filtering of the merged peptide ID lists based on sample replicate and RT‐RSD thresholds (Figure 3a). The remaining threshold values for each peptide are then averaged across replicate experiment, and filtered using the remaining threshold parameters. Peptides are then ranked by PLGS score, and duplicates removed, leaving only the top scoring peptide ID per sequence. By doing so, a list containing predictions for how many unique peptides have been identified by each trendline is provided to indicate which trendline(s) performed best. A peptide ID list of only the top‐performing trendline is then saved.

#### 2.1.3 Step 2: Merge and filter data

**FIGURE 4:**
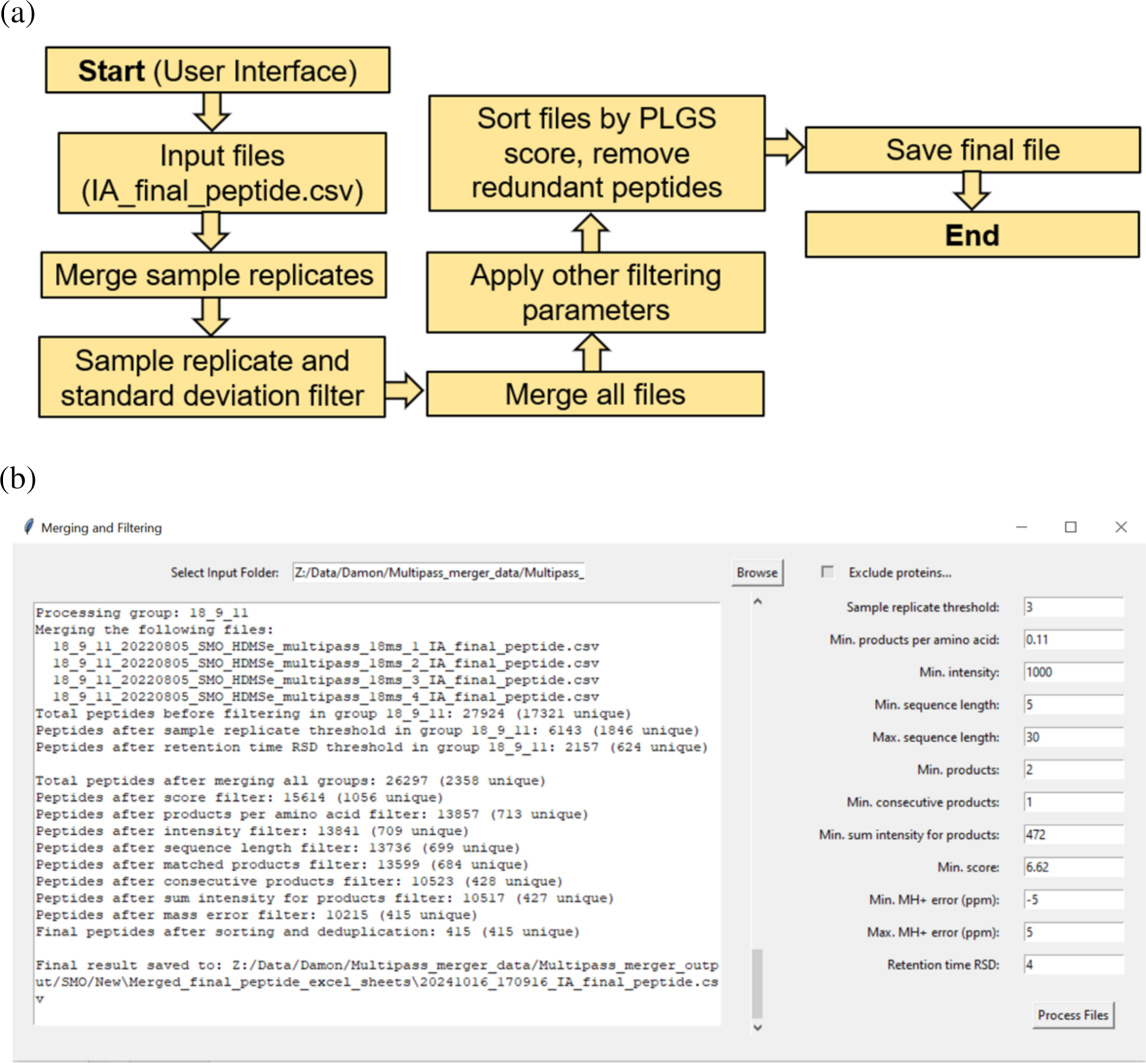
Overview of the merge and filter function. The code logic (a) and user interface (b) for “Step 2: Merge and Filter Data.” The purpose of this tool is to merge and subsequently filter all identified peptides across single‐ and multipass cIM‐HDMS^E^ experiments into a single peptide ID list. Peptide lists are merged per sample replicate, replicate threshold and RT‐RSD filters are applied, and then everything is merged into a single file. Following this, all other filtering parameters are applied, peptides are ranked by their PLGS score, and only the highest performing unique ones across the included datasets are kept.

To automate our previously published multi‐sequence cIM‐HDMS^E^ peptide mapping approach (Griffiths et al. 2024), MultiPassMerger provides the “Step 2: Merge and Filter Data” function, where common and unique peptide IDs from both single‐ and multipass cIM‐HDMS^E^ peptide mapping experiments can be merged to generate larger peptide ID lists for use in subsequent HDX‐MS experiments (Figure 4). To achieve this, the user provides a folder location containing both the single‐pass cIM‐HDMS^E^ peptide ID list from processing with auto‐calculated trendline, and the multipass cIM‐HDMS^E^ peptide ID list from the “Optional: Best Trendline Identifier” function containing IDs from the best performing trendline only (Figure 4b). After the user specifies the input folder and the desired threshold filtering parameters, the “Merge and Filter Data” function merges sample replicates, to which the RT‐RSD and sample replicate thresholds are applied (Figure 4a). Then, results from the single‐ and multipass cIM‐HDMS^E^ experiments are merged, and all other user‐specified filtering parameters are applied. Peptides are then ranked by PLGS score and redundant IDs removed, leaving only the best performing unique IDs in a final peptide list. This list is then timestamped and saved in the parent directory, from where it can be subsequently imported into DynamX or a similar software to use as a peptide ID database for manual data curation.

### 2.2 Automation of new workflow: Multi‐sequence cIM‐MS with multi‐trendline data processing

In addition to multi‐sequence analysis, the merge and filter function in MultiPassMerger also permits multi‐trendline analysis, whereby peptide ID results obtained across all of the different trendlines applied in the initial data processing step are merged to generate even larger peptide ID lists. Multi‐trendline processing can be achieved simply by skipping use of the “Optional: Best Trendline Identifier” function altogether, and instead directly providing the “Step 2: Merge and Filter” function input file location as the “Step1: Process Data” function output location. As this folder location contains the entire data processing output from the iterative trendline processing step (i.e., the single‐pass cIM peptide mapping IDs generated using auto‐calculated trendline and the multipass cIM peptide mapping IDs obtained across the use of multiple trendlines), the merge and filter function combines all data together (instead of just the single‐ and multipass peptide IDs from use of auto‐calculated and a single optimized trendline, respectively), filters via the specified thresholds, and then removes duplications to keep only the best performing ID per peptide sequence. The advantage of this approach is that, while trendline optimization can provide a single trendline that best compromises between under‐ and over‐smoothing of IM peak features across the DT versus DT FWHM distribution, the nonlinearity of multipass cIM data means that any given trendline is still unlikely to appropriately smoothen all IM features simultaneously. Consequently, during the development of MultiPassMerger, we hypothesized that each trendline would likely also contain unique peptide populations (owing to optimal smoothing conditions for different IM features under different trendlines), and that merging of results obtained across all trendlines would further increase peptide IDs. Here, we demonstrate the ability of this approach to increase peptide ID count beyond use of multi‐sequence cIM‐HDMS^E^ alone, without any increase in manual input by the user. The underlying principle of multi‐trendline processing is further explained and visualized in Data S1.

## 3 RESULTS AND DISCUSSION

### 3.1 Peptide mapping results from MultiPassMerger processing

**FIGURE 5:**
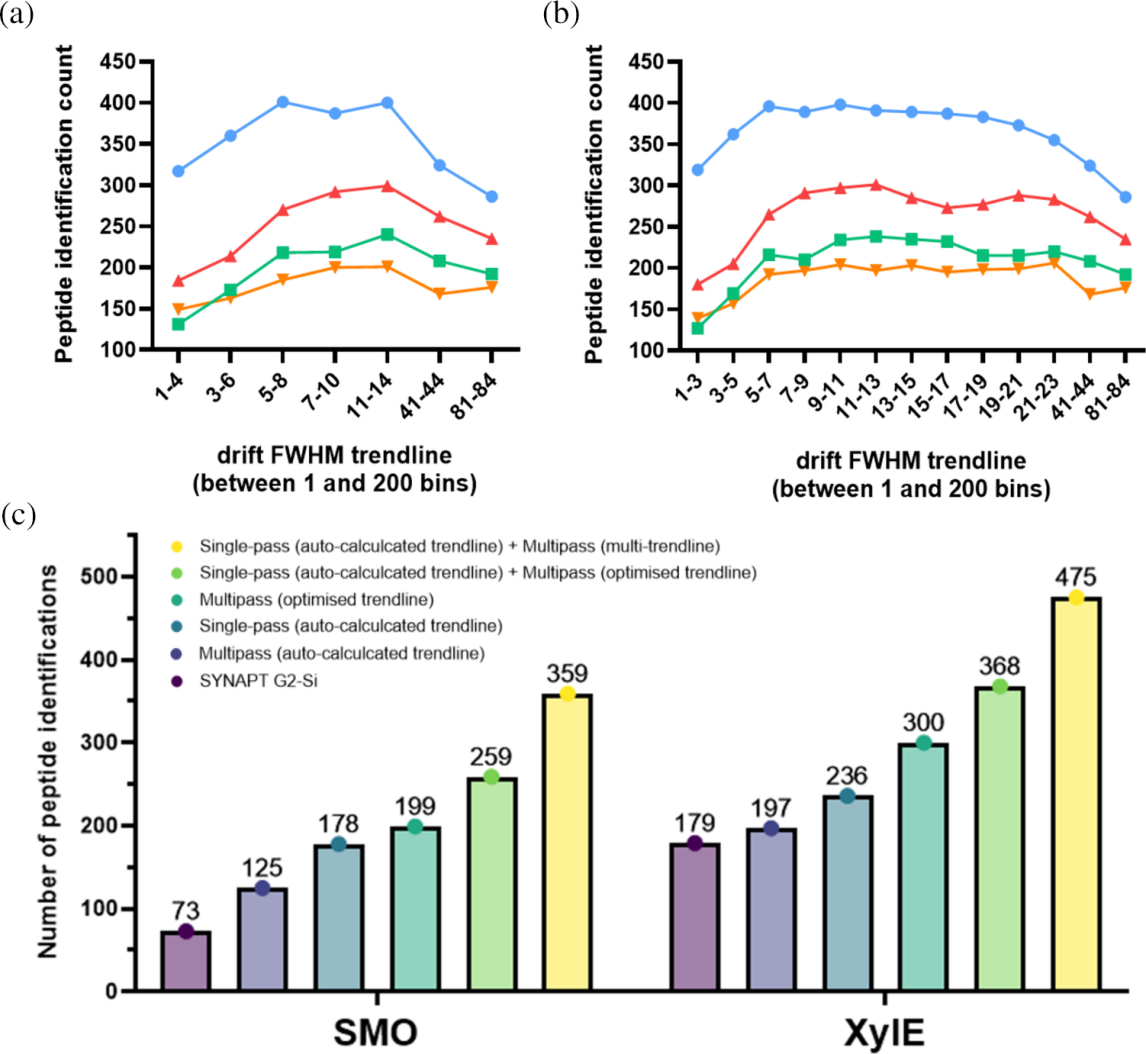
Overview of peptide mapping results from cIM‐HDMS^E^ peptide mapping analysis of model IMPs using MultiPassMerger. (a) Number of peptide IDs from multipass cIM peptide mapping of XylE, MsbA, SMO, and SecYEG using previously applied DT FWHM trendlines (Griffiths et al. 2024). (b) Number of peptide IDs in multipass cIM peptide mapping data of XylE, MsbA, SMO, and SecYEG using additional DT FWHM trendlines. (c) Number of peptide IDs after MultiPassMerger processing and DynamX manual curation of SMO and XylE data obtained using single‐pass (auto‐calculated trendline) + multipass (multi‐trendline), single‐pass (auto‐calculated trendline) + multipass (optimized trendline), and multipass (optimized trendline). Previous data generated using SYNAPT G2‐Si, Cyclic multipass, and Cyclic single‐pass processed using auto‐calculated trendline are also displayed for comparison (Griffiths et al. 2024).

To assess its functionality, we applied MultiPassMerger for peptide ID analysis of several multipass cIM‐HDMS^E^ peptide mapping datasets that we previously published using the same data processing approach but performed manually (Griffiths et al. 2024). This included the frizzled class G protein‐coupled receptor (GPCR) SMO, the secondary active transporter XylE, the ATP binding cassette transporter MsbA, and the protein translocase SecYEG, with data being generated on both SELECT SERIES Cyclic IMS and SYNAPT G2‐Si instrumentation. Due to the practical limitations of manual data processing and handling at the time, the best performing trendline for multipass cIM‐HDMS^E^ peptide mapping was optimized only for XylE and only across 7 different trendlines. This optimized trendline was then extrapolated across all multipass cIM‐HDMS^E^ datasets in an attempt to generally improve results. MultiPassMerger was employed for data processing and trendline selection for all datasets independently, using identical trendlines as were used previously (Figure 5a) (Griffiths et al. 2024). Here, a similar profile was observed across all the proteins, which is highly characteristic for this type of analysis; whereby peptide IDs are lower when using very low (e.g., 1–4) or high (e.g., 81–84) trendlines, owing to peptide ID loss from excessive under‐ and over‐smoothing of IM peak features, respectively. Interestingly, the best performing trendline for XylE was found to be 5–8, which is in contrast to our previous work where the best performing trendline was 11–14 (Griffiths et al. 2024). This discrepancy could be owing to slight differences in the PLGS release used (3.0.2 vs. 3.0.3). Nevertheless, as expected, use of MultiPassMerger with optimized trendline provided increased peptide IDs relative to default PLGS processing with auto‐calculated trendlines (Figure 5c). Moreover, for SMO, MsbA, and SecYEG, MultiPassMerger identified the best performing trendline to be 11–14 across all 3 datasets and was, therefore, comparable to previous trendline optimization efforts via XylE (Figure 5a). In an attempt to better optimize these trendlines, the data was reprocessed using 13 alternative trendlines, with the majority being clustered closely to the 11–14 trendline (Figure 5b). Here, the best performing trendline was variable between the datasets (XylE: 9–11, SMO‐BRIL: 11–13, MsbA: 11–13, SecYEG: 21–23), thereby highlighting the ability of MultiPassMerger to provide more accurate trendline optimization on a protein‐specific basis and in a fully automated fashion.

**FIGURE 6:**
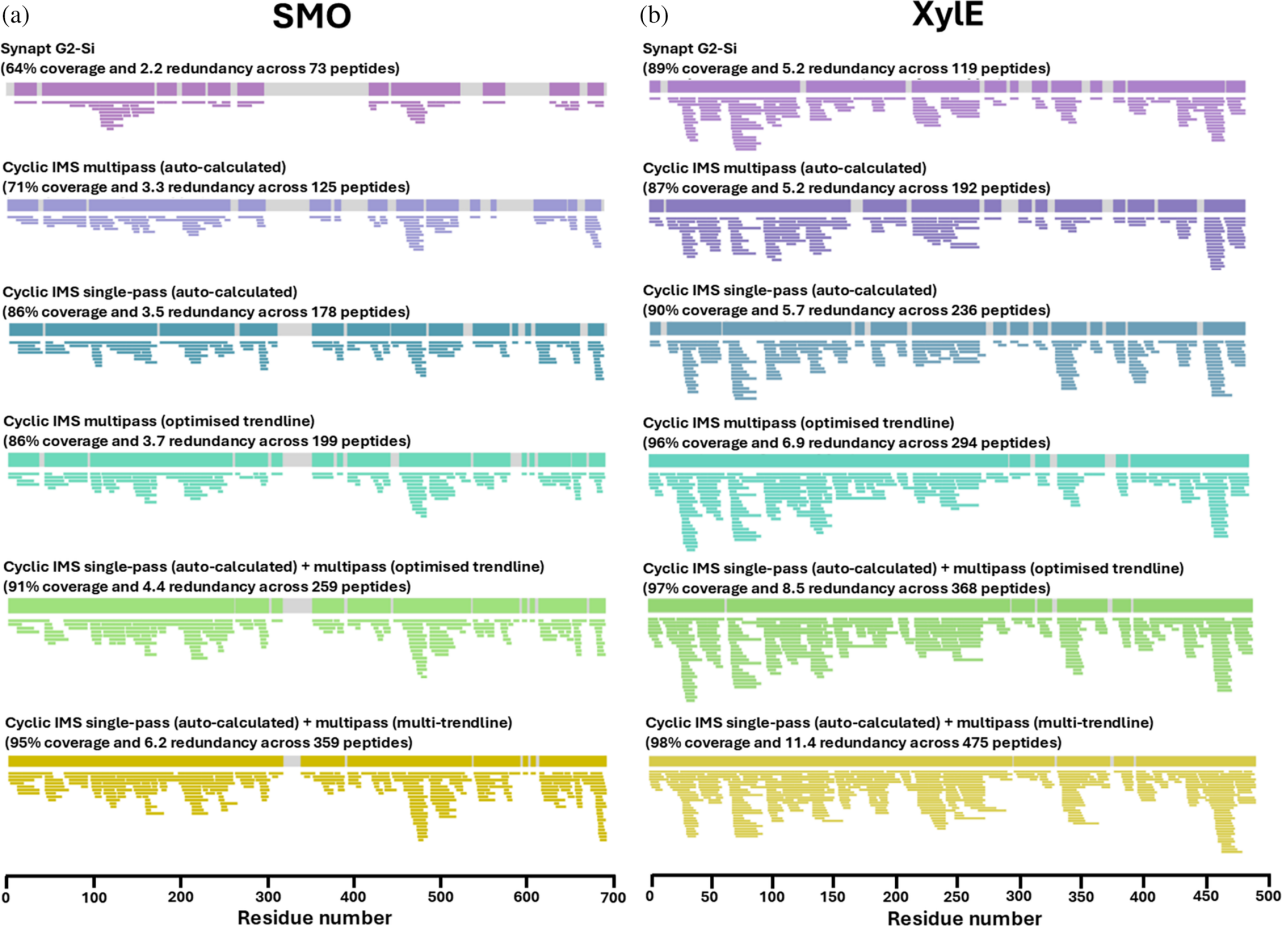
Overview of peptide coverage maps of model IMPs from cIM‐HDMS^E^ peptide mapping analysis using MultiPassMerger. Peptide coverage maps for SMO (a) and XylE (b) after MultiPassMerger processing and DynamX manual curation. Data was obtained using Cyclic IMS with single‐pass (auto‐calculated trendline) + multipass (multi‐trendline), single‐pass (auto‐calculated trendline) + multipass (optimized trendline), and multipass (optimized trendline). Previous data generated using SYNAPT G2‐Si, Cyclic IMS multipass, and Cyclic IMS single‐pass processed using auto‐calculated trendline are also displayed for comparison (Griffiths et al. 2024). The color scheme used here is identical to that used in Figure 5c.

To assess the improvement in peptide mapping data quality provided by MultiPassMerger, using the same multipass cIM‐HDMS^E^ peptide mapping data of SMO and XylE, we compared results obtained using data processing with optimized trendlines via MultiPassMerger with results obtained using the “default” PLGS workflow with auto‐calculated trendlines (Figure 5c). Here, the SMO and XylE peptide ID counts increased from 125 and 197 (when using the default PLGS workflow) to 199 and 300 (when using trendlines optimized via MultiPassMerger), respectively (Figure 5c). This also translated into increased sequence coverage and redundancy for both targets; SMO increasing from 71% coverage and 3.3 redundancy to 86% and 3.5, and XylE increasing from 87% coverage and 5.2 redundancy to 96% and 6.9 (Figure 6). As such, these results demonstrate the capability of MultiPassMerger to offset issues related to cyclic wrap‐around in multipass cIM‐HDMS^E^ peptide mapping data and increase data quality to levels comparable to, or even better than, use of single‐pass cIM‐HDMS^E^ peptide mapping with auto‐calculated trendlines (Figures 5c and 6). Once the single‐ and multipass pass cIM‐HDMS^E^ peptide mapping data were processed using the auto‐calculated and optimized trendlines, respectively, the merge and filter function in MultiPassMerger was employed to autonomously pool the results for a “multi‐sequence” peptide mapping approach, as previously described (Griffiths et al. 2024). The combination of common and unique peptide populations from each peptide mapping experiment resulted in even further increases in SMO and XylE peptide ID counts to 259 and 368 (Figure 5c), respectively, which translated into further increases in sequence coverage and redundancy to 91% and 4.4 for SMO and 97% and 8.5 for XylE (Figure 6). Crucially, the overall trends observed in these results closely mirror our previously published findings which relied on manual processing, filtering, and merging of the data (Griffiths et al. 2024). Consequently, these results highlight the capability of MultiPassMerger to markedly enhance peptide identification via multi‐sequence analysis, delivering a fully automated workflow that eliminates the labor‐intensive manual data handling efforts that this approach previously required.

As MultiPassMerger offers a means to effortlessly pool different peptide ID lists together into a single merged database, we attempted to further exploit this feature by pooling the different peptide ID lists generated by the process data function using different trendlines (i.e., as opposed to using a single optimized trendline for multipass cIM‐HDMS^E^). We hypothesized that this “multi‐trendline” approach would offer even further increases in peptide ID from the multipass cIM‐HDMS^E^ peptide mapping datasets, owing to each trendline being optimal for appropriate smoothing of different peptide populations throughout their nonlinear DT versus DT FWHM distributions. As predicted, use of MultiPassMerger to perform multi‐sequence analysis with multi‐trendline processing resulted in a significant increase in SMO and XylE peptide IDs up to 359 and 475, respectively (Figure 5c). Moreover, the increased peptide identification afforded by multi‐trendline processing also translated into further increases in sequence coverage and redundancy: 95% coverage and 6.2 redundancy for SMO and 98% coverage and 11.4 redundancy for XylE, thereby providing more information content and at better spatial resolution, far beyond what was previously obtained (Figure 6). For example, when compared to use of SYNAPT G2‐Si instrumentation with auto‐calculated trendlines under identical HDX‐MS conditions, our multi‐sequence approach on Cyclic IMS instrumentation with multi‐trendline processing via MultiPassMerger provided a SMO peptide ID increase of 392%, resulting in a 31% increase in sequence coverage and a 181% increase in redundancy. Moreover, when compared to use of Cyclic IMS with standalone single‐pass cIM and default PLGS processing, this approach increased SMO peptide ID, sequence coverage, and redundancy by 102, 9, and 68%, respectively.

It is important to highlight that the values presented above reflect peptide mapping results following curation/validation of the data in DynamX, where false positives and/or low‐quality peptides are manually removed. For SMO, manual curation of the single‐pass (auto‐calculated) + multipass (multi‐trendline) peptide mapping data led to the removal of only 13.5% of peptides from the raw identification list after filtering (Table 1). This indicates that the previously optimized threshold value (Sørensen and Salbo 2018) applied here was effective in eliminating the vast majority of false positives and/or low‐quality IDs prior to manual curation of the data. In contrast, manual curation of the XylE single‐pass (auto‐calculated) + multipass (multi‐trendline) data resulted in the removal of 27% of peptides, highlighting a higher rate of false positives and/or low‐quality peptide IDs passing the initial threshold filtering parameters prior to manual curation. This suggests that, for this type of iterative multi‐trendline processing, the previously optimized thresholds may no longer be ideal in all cases (Sørensen and Salbo 2018), and that further refinement of stricter thresholds could be beneficial. Nevertheless, the final curated peptide mapping results underscore the potential of Cyclic IMS instrumentation, coupled with MultiPassMerger processing, to significantly enhance results in HDX‐MS and other applications using cIM‐HDMS^E^ for peptide identification.

**TABLE 1:** Number of peptides, sequence coverage, and redundancy for SMO and XylE using multi‐sequence cIM peptide mapping and multi‐trendline processing.

	SMO			XylE		
	Number of peptide	Sequence coverage (%)	Sequence redundancy	Number of peptide	Sequence coverage (%)	Sequence redundancy
Raw PLGS database (uncurated)	415	97	7.19	647	100	15.95
Peptide mapping (curated)	359	95	6.20	475	99	11.28
HDX‐MS (curated)	305	94	5.25	414	99	10.07

*Note*: Values are shown for the uncurated raw PLGS database, after manual curation of the peptide mapping data, and after manual curation of the 1 min HDX timepoint data.

### 3.2 HDX‐MS analysis using peptide maps from MultiPassMerger

Owing to the increased complexity of deuterated isotopic distributions, it is common for identified peptides to be removed during HDX‐MS analysis as they cannot be reliably monitored across deuterium labelling timepoints. As such, it is important to manually validate peptides under deuterated condition to ensure accurate and reliable HDX measurements. Our previous work demonstrated that, in most cases, peptides uniquely identified in either single‐pass or multipass cIM‐HDMS^E^ experiments remain trackable in subsequent HDX measurements conducted using the converse cIM sequence (Griffiths et al. 2024). This is because the DynamX software used here for manual curation relies solely on the *m*/*z* and RT values from the peptide ID list to locate precursor ions within HDX measurement data. As long as the peptide *m*/*z* and RT remain stable between peptide mapping (e.g., identified using multipass cIM) and exchange measurements (e.g., monitored using single‐pass cIM), variations in DT values arising from use of different cIM sequences do not prevent DynamX from detecting precursor ions in the HDX measurement. Nor do DT variations hinder the generation of processed spectra based on DT extraction, as DynamX does this based on the DT value of the HDX measurement instead of the DT value in the peptide ID list itself. Therefore, while complementary single‐pass and multipass cIM‐HDMS^E^ experiments can be used to maximize peptide IDs during peptide mapping, HDX measurements only need to be performed using either standalone single‐pass or multipass cIM, not both. This suggests that, rather than better resolving precursor ions in IM space, the advantage of multi‐sequence cIM is primarily associated with enhanced precursor‐product matching, and also has the advantage of minimizing the number of LC‐MS replicates needed to generate a “complete” HDX‐MS dataset.

**FIGURE 7:**
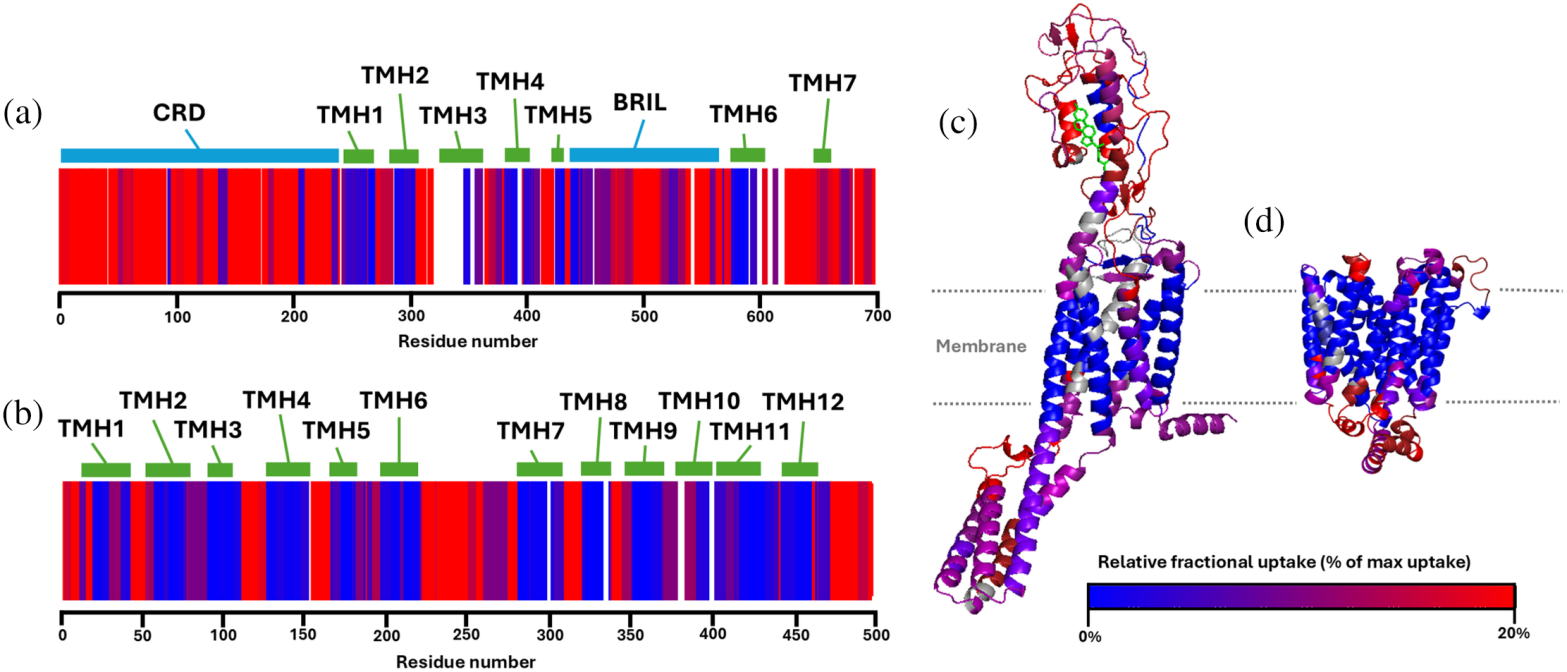
Overview of 1 min HDX‐MS timepoint data from SMO‐BRIL and XylE analyses. Heat maps of 1 min timepoint (0–20% of relative fractional uptake) from SMO (a) and XylE (b). A corresponding domain map is shown including the cystine rich domain (CRD), the transmembrane helices (TMH) and BRIL fusion protein of SMO and the TMHs of XylE. (c) Homology model of SMO (generated using PDB template: 5L7D) heat mapped with 1 min HDX timepoint. (d) Homology model of XylE (generated using PDB template: 4JA4) heat mapped with 1 min HDX timepoint.

To ensure that the additional peptide IDs provided by MultiPassMerger with multi‐trendline processing were of high quality and trackable in HDX measurements, the SMO and XylE single‐pass (auto‐calculated) + multipass (multi‐trendline) peptide maps were used to track deuteration in a 1 min HDX‐MS timepoint captured using standalone single‐pass (SMO) or multipass (XylE) cIM‐MS. Of the total 834 peptides assessed, 719 (86%) were successfully monitored (Table 1), thereby demonstrating that the proposed multi‐sequence/multi‐trendline approach not only provides increased peptide IDs but also provides high‐quality peptides that remain monitorable in subsequent HDX measurements. This is supported by the strong correlation between the observed HDX‐MS profile and known IMP transmembrane topology, which is expected owing to high protection from exchange owing to low solvent accessibility for residues within the detergent‐micelle belt (Figure 7). Importantly, of the 115 peptides removed after manual curation of the SMO and XylE data, only 62 (54%) originated uniquely from multisequence analysis with multi‐trendline processing, while the remaining 53 (46%) had already been identified using multi‐sequence with a single optimized trendline. This suggests no clear removal bias against peptides uniquely originating from multi‐trendline analysis, further reinforcing that it can provide additional high‐quality peptides that improve data quality in subsequent HDX measurements. Consequently, using single‐pass (auto‐calculated) + multipass (multi‐trendline) peptide maps maintained high effective sequence coverage after deuteration for both SMO (94%) and XylE (98%) (Table 1), in contrast to our previous multi‐sequence analyses with a single optimized trendline which led to a decline in coverage to 85% and 96%, respectively, upon deuteration (Griffiths et al. 2024). Similarly, redundancy remained high after deuteration with multi‐trendline processing (5.25 for SMO, 10.07 for XylE) (Table 1), whereas the use of multi‐sequence with a single optimized trendline saw reductions to 4.2 and 7.3 (Griffiths et al. 2024). Overall, these results highlight that multi‐trendline processing can not only increase the number of peptide IDs during peptide mapping but also increase the number of monitorable peptides in subsequent HDX‐MS experiments. This is crucial, as increased peptide content in the deuterated case is ultimately the purpose for improving peptide identification in HDX‐MS applications; leading to greater insights from deuterium incorporation in the case of sequence coverage and at greater resolution in the case of redundancy.

## 4 CONCLUSIONS AND FUTURE OUTLOOK

Previously, we demonstrated that HDMS^E^ peptide mapping experiments could be enhanced by utilizing Cyclic IMS instrumentation with multipass cIM capabilities (Griffiths et al. 2024). This was achieved through a “multi‐sequence” cIM‐HDMS^E^ approach that involved performing two complementary peptide mapping experiments: one using a single‐pass cIM sequence and the other using a multipass sequence. To address challenges associated with cyclic wrap‐around, the multipass cIM‐HDMS^E^ peptide mapping data were also processed using user‐optimized DT versus DT FWHM trendlines. The processed results were then merged with single‐pass outputs to generate larger peptide ID databases for use in subsequent HDX‐MS experiments. Despite its effectiveness, this approach was limited by the substantial manual input required during data processing. This made the workflow time‐consuming, labor‐intensive, and impractical for routine use. To overcome these limitations, we have developed MultiPassMerger, a PLGS‐based software tool designed to automate the processing, merging, and filtering of multi‐sequence cIM peptide mapping data. By streamlining these steps, MultiPassMerger simplifies this experimental approach, making it more accessible to the broader HDX‐MS community.

As the automation provided by MultiPassMerger eliminated human time constraints, we re‐analyzed previously acquired data with a wider array of trendlines, thereby allowing improvement in DT versus DT FWHM trendline optimization on a protein‐specific basis. This resulted in higher sequence coverage and redundancy for several model IMP targets during multi‐sequence cIM‐HDMS^E^ peptide mapping. Moreover, MultiPassMerger supports a “multi‐trendline” processing approach, where multiple DT versus DT FWHM trendlines can be applied iteratively to the same multipass cIM peptide mapping data, and the results are merged. This approach ensures more effective smoothing of ions across the entire DT versus DT FWHM distribution, avoiding the compromises of under‐ or over‐smoothing inherent to single‐trendline methods. Without requiring additional manual effort, the use of MultiPassMerger with multi‐trendline processing led to a significant increase in the number of peptides identified for the model IMPs SMO and XylE. Specifically, peptide IDs were up to 392% higher compared to SYNAPT G2‐Si instrumentation with linear IM and up to 102% higher than the use of Cyclic IMS instrumentation with single‐pass cIM alone. As such, whilst MultiPassMerger is capable of automating our previous multi‐sequence cIM‐MS approach with optimized trendlines (Griffiths et al. 2024), we recommend adopting a multi‐trendline approach via MultiPassMerger to provide further improvement in peptide ID. These findings demonstrate that MultiPassMerger not only improves cIM‐HDMS^E^ peptide mapping performance but also offers a user‐friendly interface that can facilitate broader adoption by the wider HDX‐MS community.

Whether employing a multi‐trendline or an optimized trendline approach, a current limitation of MultiPassMerger is the requirement for iterative processing of the same dataset, which leads to longer processing times compared to default PLGS processing. It has previously been demonstrated that use a combination of zero‐, single‐, and multipass cIM‐MS experiments can be applied to “unwrap” cIM‐MS data and allow calculation of ion periodic DT (Breen et al. 2022). By using periodic DT (how quickly the ion traverses the cIM cell once) as opposed to absolute DT (the time at which it hits the detector), this approach could potentially be adopted in the future to re‐linearize the DT versus DT FWHM relationship, thereby allowing more appropriate smoothing using a single auto‐calculated trendline. However, the tools required to implement this strategy effectively are unavailable using the current PLGS release. Nevertheless, MultiPassMerger offers a robust solution, as it automates this iterative data processing approach without requiring frequent human intervention. As such, we anticipate that MultiPassMerger will prove valuable not only for HDX‐MS but also for other structural proteomics workflows that use HDMS^E^ for peptide identification, thereby enabling a wider use of cIM‐MS technology.

## AUTHOR CONTRIBUTIONS

**Vanessa Duerr:** Investigation; methodology; validation; visualization; writing – original draft; writing – review and editing; software. **Damon Griffiths:** Conceptualization; methodology; validation; investigation; data curation; project administration; visualization; writing – review and editing; writing – original draft; supervision; formal analysis. **Argyris Politis:** Funding acquisition; resources; project administration; supervision; writing – review and editing; conceptualization.

## Supporting information


**Data S1.** Supporting Information.

## Data Availability

The data that support the findings of this study are openly available in PRIDE at https://www.ebi.ac.uk/pride/archive/projects/PXD048293, reference number Project PXD048293. MultiPassMerger is free of charge and accessible via the Politis group website (https://politislab.uk/multipassmerger). The source code is freely available on GitHub (https://github.com/vanessaduerr/MultiPassMerger). All mass spectrometry data is deposited in the ProteomeXchange Consortium via the PRIDE partner repository (Perez‐Riverol et al. 2021) with the identifier PXD048293. Other data are available upon reasonable request.
