# Supplementary material for: MultiPassMerger: Automated data processing for multipass cyclic ion mobility HDX‐MS
Source: Protein Sci. 2025 May 24;34(6):e70168. doi: 10.1002/pro.70168 (PMC12102732; doi:10.1002/pro.70168)
Supplement: Supplementary file 1 — Data S1. Supporting Information. [file PRO-34-e70168-s001.docx]

**Supplementary materials**

**Contents**

[**1.** **– Underlying principles** 2](#_Toc194489246)

[**1.1 – Linear DT vs DT FWHM relationship in typical TWIMS experiment** 2](#_Toc194489247)

[**1.2 – DT vs DT FWHM delinearization via cyclic wrap-around** 3](#_Toc194489248)

[**1.3 – Better sampling of ions in DT vs DT FWHM space using multiple trendlines** 4](#_Toc194489249)

[**2.** **– Instructions on how to use MultiPassMerger** 7](#_Toc194489250)

[**2.1 - Dependencies** 7](#_Toc194489251)

[**2.2 - Acquiring data for MultiPassMerger** 7](#_Toc194489252)

[**2.3 - How to set up multipass data acquisition in MassLynx:** 8](#_Toc194489253)

[**2.4 - Processing data using MultiPassMerger** 8](#_Toc194489254)

[**2.4.1 - Step 1: Process data** 8](#_Toc194489255)

[**2.4.2 - Step 2: Merge and Filter** 9](#_Toc194489256)

[**2.4.3 - Optional: INFO: Best trendline identifier** 9](#_Toc194489257)

[**3.** **– Experimental Methods** 10](#_Toc194489258)

[**3.1 - Creating MultiPassMerger** 10](#_Toc194489259)

[**3.2 - Materials** 10](#_Toc194489260)

[**3.3 - Peptide mapping and HDX sample handling** 10](#_Toc194489261)

[**3.4 - LC-MS with Cyclic Ion Mobility** 11](#_Toc194489262)

[**3.5 - LC-MS with linear ion mobility** 12](#_Toc194489263)

[**3.6 - Data analysis and visualization** 12](#_Toc194489264)

[**4.** **– Supplementary References** 14](#_Toc194489265)

# **– Underlying principles**

## **1.1 – Linear DT vs DT FWHM relationship in typical TWIMS experiment**

Owing to diffusion, larger ions with longer DTs tend to exhibit wider DT distributions when compared to their more compact or smaller counterparts in TWIMS experiments using drift cells of linear geometry. Consequently, when plotting ion DT FWHM as a function of DT, strong positive linear correlations are observed with high R^2^ values ^[1]^. Because it operates under the same fundamental principle, single-pass cIM experiments also exhibit this characteristic (**Supplementary figure 1**). We previously demonstrated this by plotting DT vs DT FWHM for detected peaks in several different LC-cIM-HDMS^E^ peptide mapping experiments ^[2]^. The importance of this relationship derives from the manner by which the Apex3D algorithm in PLGS smoothens peak features in IM space in both the low and high energy channels of HDMS^E^ data prior to peak detection. It does this to remove noise and generate well defined IM peaks; the point of which is to enhance DT-assisted alignment of precursors and their associated products leading to more efficient precursor-product matching in the complex DIA datasets.

In short, prior to peak detection, Apex3D samples a small number (typically around 700) extracted ion mobiligrams from the HDMS^E^ data and auto-calculates a DT vs DT FWHM trendline using an in-built linear regression model. The trendline generated here is then subsequently used to interpolate approximate peak widths for all other IM peaks in the dataset based on their DT. This step is crucial, as Apex3D uses these interpolated peak widths to select an appropriate bandwidth for kernel smoothing of each IM peak feature (**Supplementary figure 2b**). Failure to select an appropriate kernel smoother can result in ‘under-smoothing’ of the IM peak if too narrow a kernel smoother is selected and ‘over-smoothing’ if too wide a kernel smoother is selected; both of which can, in turn, result in IM peak feature loss owing to peak splitting and adjacent peak merging, respectively. As a result of IM peak feature loss, peptide IDs are reduced, as precursor and/or product ions from peptides and not appropriately included in the subsequent database search. However, owing to the strong positive linear correlation between DT and DT FWHM, a single auto-calculated trendline is normally sufficient to provide appropriate smoothing of most IM features in linear and single-pass cIM experiments, as the vast majority fall within close proximity of the calculated trendline (**Supplementary figure 2b**). As a result, the interpolated peak widths generated by the auto-calculated trendline are representative of the underlying dataset in the vast majority of cases, resulting in appropriate kernel smoothing.

## **1.2 – DT vs DT FWHM delinearization via cyclic wrap-around**

In the case of multipass cyclic cIM, ‘wrap-around’ phenomenon can occur, where slower ion populations are overtaken by speedier ions within the cIM device over multiple passes ^[3]^. Thus, depending on the geometric positioning of each ion population when ion ejection from the device is triggered, it is possible for slower ions to exit the device ahead of speedier ions. For example, this can occur when faster ions have passed the T-wave array and slower ions are behind it at the time ion ejection is triggered. Here, the slower ion is closer to the T-wave array so is ejected first, followed by the faster ion which must travel around the device an additional time before being ejected. Thus, the slower ion hits the detector first resulted in a faster apparent DT (**Supplementary figure 1a**). As a result, the linear positive DT vs DT FWHM relationship breaks down, as ion populations with any DT FWHM can be found in any DT bin ^[2]^. This often prevents Apex3D from auto-calculating a single trendline capable of appropriately smoothing all IM features, as many are positioned far below or above the trendline resulting in their under- and over-smoothing, respectively (**Supplementary figure 1c**). This, in turn, results in fewer peptide IDs owing to IM feature loss in both the low and high energy channels.

To alleviate this issue, we previously demonstrated that manual optimization of trendlines can be used to minimize peptide ID loss from over- and under-smoothing of IM features in HDMS^E^ data (**Supplementary figure 2**) ^[2]^. This can be achieved by overriding the auto-calculated trendline with a manually set trendline via the “driftFWHM-start” and “driftFWHM-end” input arguments during Apex3D processing. These variables define the applied trendline by determining the DT FHWM trendline value at DT bin 1 (driftFWHM-start) and the DT FWHM trendline value at DT bin 200 (driftFWHM-end). As such, several different trendlines can be applied to the process the same data iteratively until an optimal trendline is identified which provides the highest number of peptide identifications by best compromising between under- and over-smoothing of IM features in the data ^[2]^. We previously established a shorthand notation for describing the trendline being applied, where the driftFWHM-start and -end values are hyphen separated (i.e, an “11-13” trendline refers to a trendline with DT FWHM of 11 at DT bin 1 and DT FWHM of 13 at DT bin 200). When using multipass cIM with optimized trendlines, peptide identification improved to levels comparable to single-pass cIM processed with auto-calculated trendlines in some cases. This trendline selection and optimization to improve smoothing of IM features forms the basis of our alternative PLGS-based data processing strategy for multipass cIM data, which is fully automated by MultiPassMerger.

## **1.3 – Better sampling of ions in DT vs DT FWHM space using multiple trendlines**

Whilst trendline optimization can provide a single trendline that best compromises between under- and over-smoothing of IM features in multipass cIM data, the cyclic wrap-around-derived delinearization of the DT vs DT FWHM relationship means that any given trendline is still unlikely to appropriately smoothen all IM features simultaneously (**Supplementary figure 1c**). As a result, any single trendline is still likely to fail to capture all possible peptide identifications available in the data, owing to some IM peak features being lost. Consequently, during the development of MultiPassMerger, we hypothesized that each individual trendline applied during trendline optimization would also likely contain unique peptide populations themselves, owing to optimal smoothing conditions for different IM features under each trendline (**Supplementary figure 2**), and that merging peptide identification results obtained across all the screened trendlines would further increase peptide IDs. Here, we term this strategy ‘multi-trendline’ analysis, which can be used in conjunction with the multi-sequence analysis approach to further increase the number of high-quality peptide identifications made. Consequently, more peptides pass the set filtering thresholds in DynamX, leading to better data quality at both the peptide mapping and HDX-MS levels. MultiPassMerger also features the ability to fully automate multi-trendline processing in addition to automating our previous workflow using singular optimized trendlines.


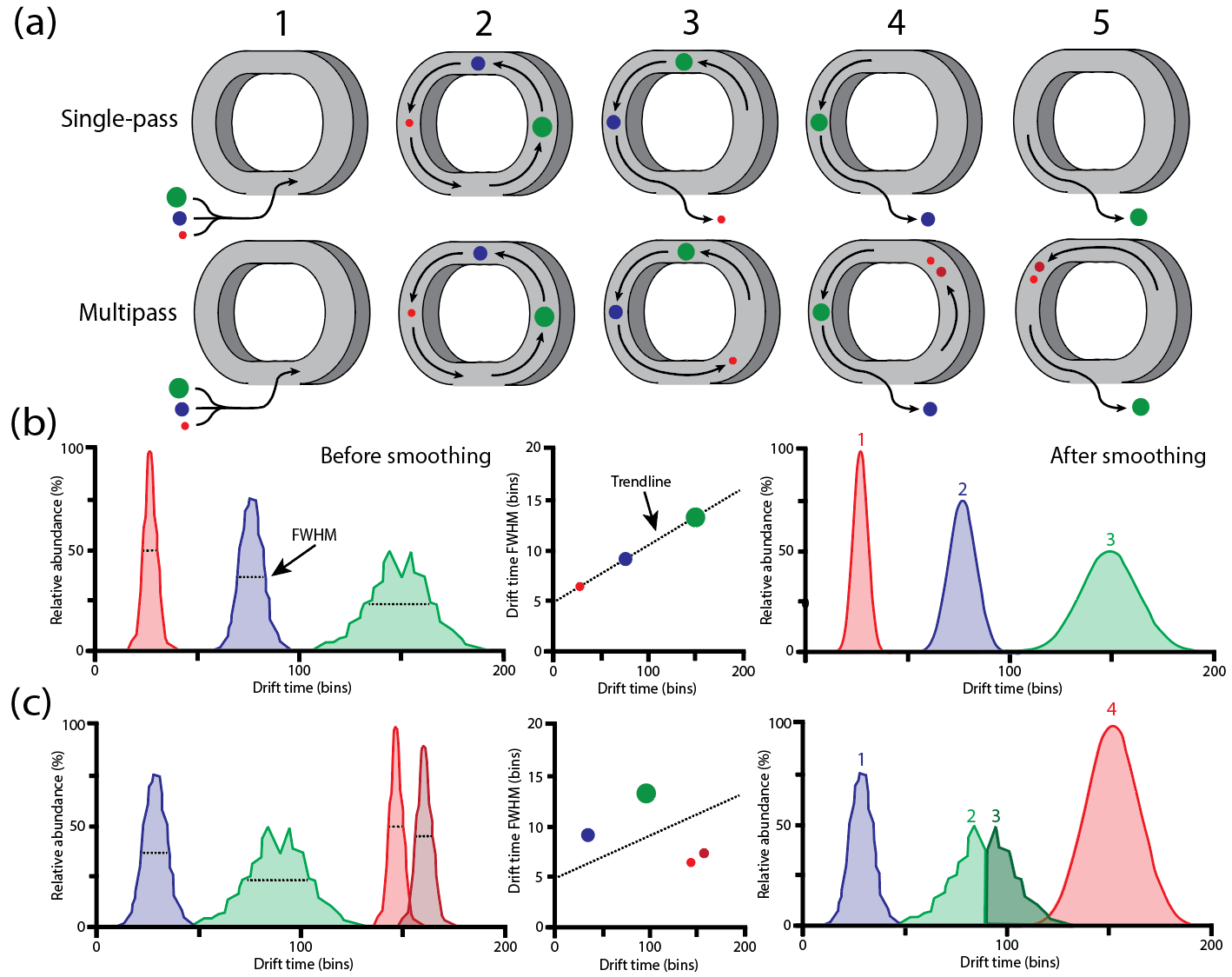


**Supplementary figure 1. Illustration of DT vs DT FWHM delinearization via cyclic wrap-around and IM peak feature loss from improper kernel smoothing. (a)** In single-pass cIM, three +1 charge ions — small (red), intermediate (blue), and large (green) — enter the device (1), separate by size/shape (2), and exit in order: red (3), blue (4), green (5). In multipass cIM, ions enter (1), separate (2), but the red ion wraps around (passes the T-wave array) before ejection is triggered (4). Enhanced IM resolution reveals two red ion species, causing the blue to exit first (4), followed by the green (5), and then the two red ions later. **(b)** Single-pass cIM produces a mobiligram with three peaks and a strong DT vs DT FWHM correlation with a high R² trendline. As all ions align closely to the trendline, appropriate kernel bandwidths are selected yielding well-defined peaks. **(c)** Multipass cIM generates four peaks for the blue, green, and two red ions. The blue and green ions eject first, disrupting the linear DT vs DT FWHM correlation. The red ions fall well below the trendline are, thus, over-smoothed, resulting in IM feature loss from peak merging, while the green ion IM feature above the trendline is under-smoothed, causing IM feature loss from peak splitting by the retained noise.


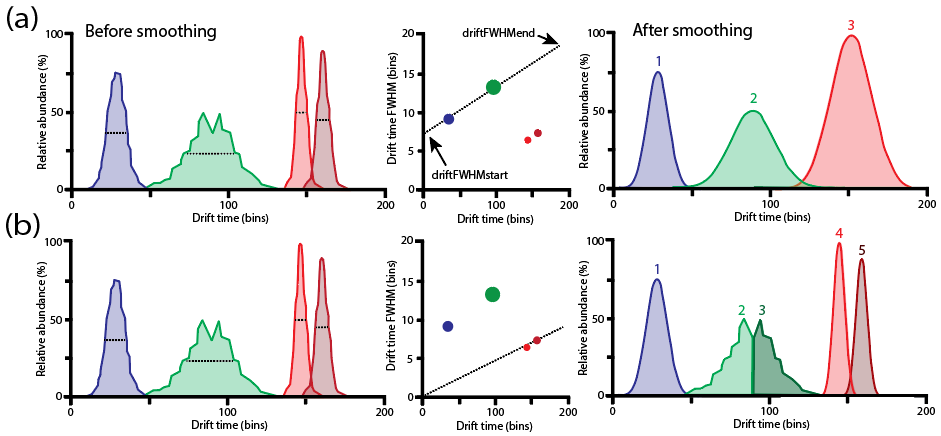


**Supplementary figure 2. Illustration of improved kernel smoothing via manual trendline adjustment.** Here, illustrations of mobiligrams (before and after smoothing) and DT vs DT FWHM trendlines are shown from the multipass cIM separation of the red, blue and green ions in **Supplementary Figure 1a**. **(a)** To prevent under-smoothing of the IM features of the blue and green ions, the trendline is manually shifted upward (using the driftFWHM-start and -end values) to intersect with the FWHM values for the blue and green ions. Consequently, when performing kernel smoothing, accurate measurements of DT FWHM are used leading to more appropriate smoothing, which prevents IM feature loss from splitting of the green ion peak. However, over-smoothing still occurs to the red ion IM features resulting in IM feature loss **(b)** To prevent over-smoothing of the IM features of the red ions, the trendline is manually shifted downward to intersect with the red ions. This prevents IM feature loss from adjacent peak merging of the two red ion IM peaks by proving more accurate measurements of their DT FWHM. However, under-smoothing still occurs to the IM feature of the green ion, resulting in IM feature loss from peak splitting. It’s important to note that both trendlines result in different IM features being lost. Thus, each trendline can generate different populations of identified peptides during subsequent database searching depending on which IM features are retained. Therefore, when using a single trendline, the best performing trendline will be those that best compromise between under- and over-smoothing, whereas use of multiple trendlines can better sample all IM features across the DT vs DT FWHM distribution by processing data in an iterative manner.

# **– Instructions on how to use MultiPassMerger**

***Note: Please visit the Politis group website for a more interactive and detailed tutorial:*** <https://politislab.uk/multipassmerger>

## **2.1 - Dependencies**

Software requirements:

- MassLynx (Waters Corporation – any version)
- ProteinLynxGlobalServer (PLGS) (Waters Corporation – version 3.0.3 *)
- MultiPassMerger (Politis Laboratory – download here: <https://drive.google.com/file/d/1aAjI8ZnCpQEwDOHKl11a7j39M9bQFpsY/view> )
- Optional: DynamX (Waters Corporation – any version)

Hardware/instrument requirements:

- SELECT SERIES cIM-MS (Waters Corporation)
- A computer for processing (any)

*MultiPassMerger will look for ProteinLynxGlobalServer version 3.0.3 in your C drive. If you have an older version (e.g. 3.0.2) it will still work – simply re-name it to “3.0.3”.

## **2.2 - Acquiring data for MultiPassMerger**

Background: You have a target protein for which you want to create a high-quality peptide map, but a single-pass run with your chosen bottom-up LC-MS method did not yield good enough results. You decide to re-run your peptide map with multipass IM-MS separation and to process and merge your data using MultiPassMerger to gain additional peptides.

- One pass = 2ms cIM separation
- Multipass = Anything above 10ms. We recommend 18ms for membrane proteins, Phosphorylase B from rabbit muscle or similarly sized targets.
- Using MultiPassMerger, you can process and merge *any number of datasets* into a single peptide map
- IMPORTANT: ensure to include the IM-MS separation time in your sample names, e.g. “PhosB_18ms_OCT2024”. Otherwise MultiPassMerger won’t be able to identify sample replicates.

## **2.3 - How to set up multipass data acquisition in MassLynx:**

Open MassLynx, open the MS Tune window and open the Cyclic Sequence Control window. Select and open any previous MS^E^ ion-mobility sequence. In “Sequences”, look for “2 Separate”. Right of that, you can change the ion-mobility separation in milliseconds. Choose which IM-MS separation time you want (e.g. 18ms). Save your new sequence under a new name. Use this sequence instead of your single-pass sequence when acquiring data.

## **2.4 - Processing data using MultiPassMerger**

### **2.4.1 - Step 1: Process data**

Open “Step 1: Processing data” in the main interface of MultiPassMerger.

- Browse for your one pass and/or multipass folder(s) containing the raw data you want to process. If you leave one empty, the software will ignore it and proceed to process only the raw data you did specify.
- Change the processing parameters as desired. MultiPassMerger comes pre-filled out with suggested values, including multipass trendlines.
- Export your workflow parameters from PLGS, and select the file in MultiPassMerger
- Select a FASTA file, which contains the sequence of your target protein (and of any contaminants, e.g. pepsin)
- Choose an output folder, where your data will be saved
- Click process

MultiPassMerger will now write and execute a batch file that prompts the PLGS ion and peptide search algorithm to process your data as specified.

After several hours (depending on the number of raw data files and multipass trendlines chosen), your data will have finished processing and will be saved in the form of peptide lists (final_peptide.csv) in sub-folders in your output folder location.

One pass data will be saved in a sub folder called 1_1. Multipass data will be sorted into subfolders according to the trendlines used, e.g. 3_6, 6_9, 9_12 etc...

### **2.4.2 - Step 2: Merge and Filter**

Open “Step 2: Merge and Filter” in the main interface of MultiPassMerger.

- Select the output folder from the previous step (containing your final_peptide.csv files, sorted into sub-folders) as the input folder
- Clicking “Exclude proteins...” allows you to exclude any unwanted protein from the analysis (e.g. any contaminants you specified in your FASTA file earlier)
- On the right side, you may choose to change the filtering parameters (MultiPassMerger comes pre-filled out with suggested values)
- Click process

After a few seconds, MultiPassMerger will have created a single peptide list, containing only the single best performing unique peptides across all datasets.

If using DynamX, you can then add this peptide list together with the raw data, and, if desired, any HDX data (which can be run with one pass as normal).

### **2.4.3 - Optional: INFO: Best trendline identifier**

Open “INFO” Best trendline identifier” from the main MultiPassMerger interface if you want to see how well your chosen multipass trendlines performed. This may be good to know when you first run this method, as different acquisition settings with different targets may require different trendlines for appropriate data smoothening. In general, the more data smoothening trendlines are chosen, the better – but they will take a lot of time to process.

# **– Experimental Methods**

The data analyzed here has already been acquired and published elsewhere ^[2]^. A brief description of the overall data acquisition method will be presented here. For a detailed description please methods section in main article and supplementary materials in Griffiths et.al (2024) for a detailed description ^[2]^.

## **3.1 - Creating MultiPassMerger**

The software was developed using Python 3.12 in the IDLE shell environment for testing. The graphical user interface (GUI) was created using Tkinter. PyInstaller was utilized to package the software into an executable (.exe) file. The custom Python GPT-4 model by Nickolas Baker was employed to assist with code generation, debugging, and optimization ^[4]^.

## **3.2 - Materials**

Facade-EM detergent was purchased from Avanti Polar Lipids (Alabaster, Alabama) and *n*-dodecyl-β-d-maltopyranoside (DDM) was purchased from Anatrace (Maumee, Ohio). Unless otherwise stated, all other chemicals and reagents were purchased from Sigma-Aldrich (Gillingham, Dorset). Sample expression and purification information can be found in supporting information of Griffiths et al ^[2]^.

## **3.3 - Peptide mapping and HDX sample handling**

Sample preparation and handling were automated using a Trajan HDX PAL system (LEAP Technologies, Carrboro). For nondeuterated peptide mapping, 5 μL of protein (20 μM XylE, 8.32 μM SMO, 21.4 μM MsbA, or 4 μM SecYEG) was diluted in its respective equilibration buffer:

- **XylE**: 27.5 μL of 10 mM potassium phosphate + 0.02% DDM, pH 7.4
- **SMO**: 27.5 μL of 50 mM HEPES + 200 mM NaCl + 0.03%/0.003% DDM/CHS, pH 7.4
- **MsbA**: 95 μL of 20 mM Tris + 150 mM NaCl + 0.02% facade-EM, pH 7.4
- **SecYEG**: 95 μL of 20 mM Tris + 50 mM KCl + 0.02% DDM, pH 8

For HDX, samples were diluted in deuterated equivalents of their equilibration buffers for 1 min, then quenched (1:1) in buffer at 1 °C for 15 s:

- **XylE**: 100 mM potassium phosphate + 0.1% DDM, pH 2.3
- **SMO**: 100 mM potassium phosphate + 100 mM TCEP + 4 M urea + 0.1% DDM, pH 2.3
- **MsbA**: 100 mM potassium phosphate + 4 M urea, pH 2.3
- **SecYEG**: 0.7% formic acid + 0.1% DDM, pH 2.3

Quenched samples (60 μL for XylE and SMO, 100 μL for MsbA and SecYEG) were injected into a 50 μL loop and passed through a BEH Enzymate column (Waters Corporation) with immobilized porcine pepsin at 20 °C. Nondeuterated samples were performed in quadruplicate, and HDX samples were performed in triplicate.

## **3.4 - LC-MS with Cyclic Ion Mobility**

LC-cIM-MS experiments were conducted using a SELECT SERIES Cyclic IM QTOF coupled to an M-class nanoACQUITY UPLC and HDX manager (Waters Corporation). After digestion, peptides were trapped/desalted on a BEH C18 VanGuard precolumn at 100 μL/min for 3 min in mobile phase A (0.2% formic acid in H2O), followed by UPLC separation on a 1 mm × 100 mm BEH C18 column at 40 μL/min with an 8–55% gradient of mobile phase B (0.2% formic acid in acetonitrile) over 8 min. All chromatography was performed at 1 °C to minimize back-exchange.

Electrospray ionization operated in positive mode, with the QTOF in resolution V-mode and HDMS^E^ for data-independent acquisition. Calibration was performed with sodium iodide, and leucine enkephalin served as lock mass for post-acquisition correction. Spectra (50–2000 m/z) were acquired with capillary voltage at 3.0 kV, sample cone at 20 V, source offset at 30 V, source temperature at 100°C, desolvation gas flow at 800 L/hr, trap MS collision energy at 6 eV, and transfer CE ramping from 15–50 eV.

**Cyclic Ion Mobility (cIM):**

- *Single-pass:* 10 ms injection, 3 ms separation, 34 ms ejection/acquire; TW height: 23 V, ADC delay: 13 ms, two pushes per bin.
- *Multipass:* 10 ms injection, 18.13 ms separation, 34 ms ejection/acquire; TW height: 22 V, ADC delay: 28 ms, two pushes per bin.

To prevent carryover, the Enzymate column was cleaned with pepsin wash (1.5 M Gu-HCl, 0.4% MeOH, 0.5% formic acid, pH 3), and a sawtooth LC gradient was run between injections. All LC-MS instrument and experimental parameters were kept identical across both the non-deuterated peptide mapping and deuterated exchange measurements.

## **3.5 - LC-MS with linear ion mobility**

For experiments using linear TWIM, the method outlined above was performed but with a SYNAPT G2-Si QTOF instrument (Waters Corporation, Wilmslow). Identical UPLC equipment, buffers, samples, and columns were used to maximize comparability. Further details regarding instrument tuning and mobility parameters can be found in the supporting information of Griffiths et al ^[2]^.

## **3.6 - Data analysis and visualization**

Nondeuterated HDMS^E^ files were processed using PLGS v3.0.2 (Waters Corporation) via MultiPassMerger using thresholds of 250 and 100 for low and high energy, respectively. The digest reagent was set to nonspecific with 0 missed cleavages and no false discovery rate filter. Apex3D was operated using the following DT vs DT FWHM trendlines to repeat our previous work: 1-4, 3-6, 5-8, 7-10, 11-14, 41-44, and 81-84, and the following trendlines for the final data processing in this manuscript: 1-3, 3-5, 5-7, 7-9, 9-11, 11-13, 13-15, 15-17, 17-19, 19-21, 21-23, 41-44, and 88-84. Peak lists were searched against the target protein(s) and pepsin sequences.

For multisequence analysis using optimised trendline, MultiPassMerger was applied to identify the top performing trendline in multipass data by filtering using the following threshold parameters:

- Minimum intensity: 1000
- Sequence length: 5–30 residues
- Products: ≥2 total, ≥1 consecutive, ≥0.11 average per residue
- Sum intensity: ≥472
- PLGS score: ≥6.62
- Precursor error: ≤5 ppm
- Retention time RSD: ≤4%
- Sample replication threshold: 3/4 replicates

Once identified, MultiPassMerger was used to merge the multipass cIM peptide identification list from the analysis using the optimal trendline with the peptide identification list from single-pass analysis using auto-calculated trendline. The merged results were then filtered using the same aforementioned threshold parameters.

For multisequence analysis using multi-trendline trendline processing, MultiPassMerger was used to merge all of the peptide identification results from differential DT vs DT FWHM processing of multipass merger with the results from single pass using auto-calculated trendlines. This merged list was then filtered using the following threshold parameters:

- Minimum intensity: 1000
- Sequence length: 5–30 residues
- Products: ≥2 total, ≥1 consecutive, ≥0.11 average per residue
- Sum intensity: ≥472
- PLGS score: ≥6.62
- Precursor error: ≤5 ppm
- Retention time RSD: ≤4%
- Sample replication threshold: 3/4 replicates

For both multi-trendline and optimised trendline analyses, the final peptide lists were imported into DynamX v3.0 (Waters Corporation) and used to search for peptides in the reference raw files and measure deuterium uptake in a 1 min HDX-MS timepoint captured using either standalone single-pass (SMO) or multipass (XylE) in triplicate. Peptides were then manually validated and reviewed in DynamX. During manual curation, peptide precursor ions were only accepted if they passed the following criteria: 1) all *m/z* peaks of the isotopic distribution were visible and assigned after generation of spectra based on drift time extraction so that accurate deuterium uptake could be measured, 2) peptide precursor ions in both the reference and HDX measurements could be tracked across triplicate experiment, 3) the recorded *m/z* values of all assigned *m/z* peaks fell within the mass error ranges provided by the DynamX isotope worksheet, and 4) the recorded values of all assigned *m/z* peaks fell within 0.25 min of the RT values provided during PLGS peptide identification. If peptides did not pass the aforementioned criteria they were removed from the analysis. Manually curated data and heat maps were then exported to Deuteros v2.0 ^[5]^, Prism, and PyMOL for interpretation and visualization.

# **– Supplementary References**

(1) Kune, C.; Far, J.; De Pauw, E. Accurate Drift Time Determination by Traveling Wave Ion Mobility Spectrometry: The Concept of the Diffusion Calibration. *Analytical Chemistry* **2016**, *88* (23), 11639-11646. DOI: 10.1021/acs.analchem.6b03215.

(2) Griffiths, D.; Anderson, M.; Richardson, K.; Inaba-Inoue, S.; Allen, W. J.; Collinson, I.; Beis, K.; Morris, M.; Giles, K.; Politis, A. Cyclic Ion Mobility for Hydrogen/Deuterium Exchange-Mass Spectrometry Applications. *Analytical Chemistry* **2024**, *96* (15), 5869-5877. DOI: 10.1021/acs.analchem.3c05753.

(3) Breen, J.; Hashemihedeshi, M.; Amiri, R.; Dorman, F. L.; Jobst, K. J. Unwrapping Wrap-around in Gas (or Liquid) Chromatographic Cyclic Ion Mobility–Mass Spectrometry. *Analytical Chemistry* **2022**, *94* (32), 11113-11117. DOI: 10.1021/acs.analchem.2c02351.

(4) OpenAI. *Learning to Reason with LLMs*. 2024. <https://openai.com/index/learning-to-reason-with-llms/> (accessed 2024 12/09).

(5) Lau, A. M.; Claesen, J.; Hansen, K.; Politis, A. Deuteros 2.0: peptide-level significance testing of data from hydrogen deuterium exchange mass spectrometry. *Bioinformatics* **2021**, *37* (2), 270-272. DOI: 10.1093/bioinformatics/btaa677 (acccessed 11/27/2024).
